# Complete Genome Sequences of the Obligate Symbionts “*Candidatus* Sulcia muelleri” and “*Ca.* Nasuia deltocephalinicola” from the Pestiferous Leafhopper *Macrosteles quadripunctulatus* (Hemiptera: Cicadellidae)

**DOI:** 10.1128/genomeA.01604-15

**Published:** 2016-01-21

**Authors:** Gordon M. Bennett, Simona Abbà, Michael Kube, Cristina Marzachì

**Affiliations:** a University of Hawaii at Manoa, Department of Plant and Environmental Protection Sciences, Honolulu, Hawaii, USA; b Istituto per la Protezione Sostenibile delle Piante, CNR, Torino, Italy; c Albrecht Daniel Thaer-Institute, Humboldt-Universität zu Berlin, Berlin, Germany

## Abstract

Two bacterial symbionts of the European pest leafhopper, *Macrosteles quadripunctulatus* (Hemiptera: Cicadellidae), were fully sequenced. “*Candidatus* Sulcia muelleri” and “*Ca*. Nasuia deltocephalinicola” represent two of the smallest known bacterial genomes at 190 kb and 112 kb, respectively. Genome sequences are nearly identical to strains reported from the closely related host species, *M. quadrilineatus*.

## GENOME ANNOUNCEMENT

Insects that feed on plant-sap diets (Hemiptera) rely on obligate microbial symbionts to provide essential nutrition (1). In the Auchenorrhyncha suborder, many hosts harbor two co-primary symbionts, “*Candidatus* Sulcia muelleri” (*Bacteroidetes*) and a *betaproteobacterium*. Together, these symbionts provide insect hosts with the ten essential amino acids (EAAs) (2–4). Recent genomic analysis revealed that these symbionts have the smallest known genomes, ranging between 112 and 245 kb (2–8). Both originated with the their hosts over 260 million years ago (6, 9, 10).

We sequenced “*Ca*. Sulcia” and the betaproteobacterium, “*Ca.* Nasuia deltocephalinicola,” strains from the leafhopper *Macrosteles quadripunctulatus* (Cicadellidae: Deltocephalinae: PUNC) (9, 11). This species is a pest throughout Europe, vectoring economically important “*Candidatus* Phytoplasma” plant pathogens (12, 13). Genomes were sequenced in order to characterize bacterial symbiont populations infecting *M. quadripunctulatus*.

Ten insect specimens were pooled from lab-reared colonies collected in Torino, Italy. Genomic DNA was extracted with a CTAB protocol and RNase treatment (14). DNA was sequenced with PacBio (405,502 reads, average length 991 bp) and paired-end Illumina HiSeq (2 × 100 bp reads, 300 bp insert, ~13 million reads). Illumina-derived reads were adaptor trimmed and quality filtered in Trimmomatic v0.33 (15). PacBio reads were corrected with post-processed Illumina reads through PacBioToCA (16). *De novo* hybrid genome assemblies were done with SPAdes v3.6.1 (17). BLASTn was used to bin symbiont contigs (18). “*Ca*. Sulcia” and “*Ca*. Nasuia” were assembled into one and three contigs, respectively. Contigs were merged into single scaffolds in Geneious v9.0.2 (19). Reads were mapped iteratively to reordered symbiont scaffolds with SOAP2 v2.22 to verify consistent chromosomal coverage (20). Average read depth for completed genomes was 94-fold for “*Ca.* Sulcia” and 130-fold “*Ca.* Nasuia.” Refinement revealed a 3-fold coverage spike in “*Ca*. Nasuia” starting at nucleotide position 106,992 and corresponding to an 85 bp AT-rich, noncoding repeat. Coding content was predicted via RAST with Glimmer, and verified manually with respect to previously sequenced strains (21, 22). Pair-wise divergence for all protein-coding genes was estimated following previously described pipelines (23).

The circular chromosomes of “*Ca.* Nasuia” and “*Ca.* Sulcia” PUNC comprise 112,031 bp and 190,671 bp, respectively. The “*Ca*. Nasuia” PUNC genome is characterized by a G+C content of 16.6%, and it encodes 137 protein-coding genes (CDS), 29 tRNAs, and a 16S-23S-5S rRNA operon. It retains the metabolic pathways for two EAAs, histidine and methionine. In contrast, the “*Ca.* Sulcia” PUNC genome has a G+C content of 24.4% and encodes 190 CDS, 30 tRNAs, and a 16S-23S-5S rRNA operon. It retains the complementary pathways for synthesizing the remaining eight EAAs.

Results confirm that the leafhopper genus *Macrosteles* hosts obligate nutritional symbionts with two of the smallest known genomes (6, 8). Both PUNC symbiont genomes exhibit perfectly conserved sequence synteny and coding content in line with strains previously sequenced from *Macrosteles quadrilineatus* (ALF) (6). However, as observed in other leafhoppers, the average pair-wise divergence for protein-coding genes is lower between “*Ca.* Nasuia” PUNC and ALF strains (90.47%) than it is for the highly conserved “*Ca.* Sulcia” symbiont (99.68%) (24).

### Nucleotide sequence accession numbers.

Complete genome sequences have been deposited in DDBJ/ENA/GenBank under accession numbers CP013211 to CP013212.
